# Clinical characteristics and prognosis of SARS-CoV-2 infection in lung transplant recipients

**DOI:** 10.3389/fsurg.2024.1354994

**Published:** 2024-05-01

**Authors:** Wenping Zhang, Qiangming Li, Zeheng Ma, Zhijun Han, Shuai Hu, Tian Xia, Zibo Zhu, Li Wei

**Affiliations:** Department of Thoracic Surgery/Lung Transplantation, Zhengzhou Key Laboratory for Surgical Treatment for End-Stage Lung Disease, Henan Provincial People’s Hospital, People’s Hospital of Zhengzhou University, People’s Hospital of Henan University, Zhengzhou, China

**Keywords:** COVID-19, lung transplant recipients, immune compromised, solid organ transplantation, prognosis

## Abstract

**Objective:**

This study aimed to investigate the clinical manifestations and prognosis of lung transplant (LTx) recipients infected with severe acute respiratory syndrome coronavirus 2 (SARS-CoV-2) during the coronavirus disease (COVID-19) pandemic.

**Methods:**

The research participants were LTx recipients who underwent surgery and were regularly followed up at our center. From 1 December 2022 to 28 February 2023, during the COVID-19 pandemic in China, research participants were interviewed either online or in person. SARS-CoV-2 nucleic acid or self-tested antigens were detected according to accessibility. Diagnosis and treatment were performed according to the Diagnosis and Treatment Plan for COVID-19 (10th edition) issued by the National Health Commission of the People's Republic of China. Hospitalized patients underwent chest imaging examinations, routine blood tests, biomarkers for infection and inflammation, and biochemical tests, all of which were taken and recorded. Data were analyzed to describe the features of COVID-19 in LTx recipients.

**Results:**

In total, 52 patients were enrolled in this study, comprising 48 men and 4 women, with a mean age of 51.71 ± 11.67 years. By 1 December 2022, the mean survival period was 33.87 ± 25.97 months, of which 84.61% of the patients (44/52) had a survival period longer than 12 months. The SARS-CoV-2 infection rate in these LTx recipients was 82.69% (43/52), with 3.85% (2/52) of the infected recipients being asymptomatic, 50.00% (26/52) of the infected recipients experiencing mild COVID-19, 11.54% (6/52) having moderate COVID-19, and 17.31% (9/52) having severe or critical COVID-19. The mortality rate among severe and critical patients was 66.67% (6/9).

**Conclusion:**

LTx recipients in this cohort exhibited a notable susceptibility to SARS-CoV-2, with 82.69% of individuals diagnosed with COVID-19. Moreover, the mortality rate among critically ill patients was high.

## 1 Introduction

Solid organ transplant (SOT) recipients are among the most vulnerable human subpopulations and are at a higher risk of developing serious illness from coronavirus disease (COVID-19). Immunosuppressive treatments continue to pose serious challenges to the prevention of severe or fatal outcomes of COVID-19 (1). The available data on the incidence, clinical characteristics, and prognosis of COVID-19 in SOT recipients are limited, particularly regarding lung transplant (LTx) recipients (2–6, 11, 12). Although lung transplantation accounts for a relatively small proportion of SOT, with LTx recipients accounting for 4.48% of all SOT recipients worldwide, this proportion is projected to increase to 4.80% in China by 2021 (7).

On 7 December 2022, China adjusted its epidemic prevention and control policy, and the prevention and control of COVID-19 entered a new stage in Chinese society. Based on data from the Chinese Center for Disease Control and Prevention, the number of positive COVID-19 nucleic acid tests and the positive rate among the reporting population within all provinces initially showed an upward trend, followed by a subsequent decline since 9 December 2022. On 22 December 2022, the number of individuals testing positive for COVID-19 peaked at 6.94 million, which then fluctuated and decreased to 2,661 on 20 April 2023. Simultaneously, the positive rate of COVID-19 tests peaked at 29.2% on 25 December 2022, followed by fluctuations and subsequent decline (8). Considering the background of the pandemic's dynamics, we aimed to explore the incidence, clinical characteristics, and prognosis of COVID-19 among LTx recipients.

## 2 Methods and materials

### 2.1 Study design

This study included all LTx recipients who underwent lung transplantation surgery and regular follow-ups at the Lung Transplantation Center of Henan Provincial People's Hospital. Following 7 December 2022, all lung transplantation recipients were tested for SARS-CoV-2 antigen at home, or throat swabs were collected to test for SARS-CoV-2 nucleic acids according to the research protocol. We advised our LTx recipients to undergo testing if they exhibited symptoms, had contact with a positive case, or were due for their community's routine screening. They were to report their test results to the researchers. Follow-ups were conducted either online or in person by LTx specialists at the transplant center of Henan Provincial People's Hospital. In the event of a COVID-19 diagnosis, the patients were treated in accordance with the guidelines outlined in the Diagnosis and Treatment Plan for Novel Coronavirus Pneumonia (Tenth Edition for Trial Implementation) issued by the China National Health Commission (9).

Data pertaining to patient demographics, baseline comorbidities, transplant-related history, symptoms, focused laboratory values, and imaging data were systematically collected during the initial phase. Follow-up components included hospital and intensive care unit admissions, mechanical ventilation, death, time to death, complications, and management strategies. The study period spanned from 1 December 2022 to 28 February 2023, with a rigorous 28-day follow-up for all patients who had COVID-19. This study was reviewed and approved by the Ethics Committee of Henan Provincial People's Hospital 2021 (No. 122).

### 2.2 Statistical analysis

The patients were divided into groups according to the severity of COVID-19 (9), and the clinical parameters of the patients in each group were described (Table 1). Measurement data conforming to normal distribution are expressed as mean ± standard deviation, and the comparison between the two groups was performed using a *t*-test or a *t*'-test. The median (quartile) [M (QL, QU)] was used to present measurement data that did not conform to the normal distribution. We assessed all collected variables using univariate analysis to identify the associations between baseline covariates and COVID-19 severity. Logistic regression analysis was performed to identify the risk factors for severe-to-critical COVID-19 in LTx recipients. The primary endpoint of the study was the 28-day mortality rate of patients with COVID-19 among the LTx recipients. Variables such as interleukin 6, C-reactive protein, and arterial oxygen partial pressure (PaO_2_)/oxygen uptake concentration (FiO_2_) were included in the death risk factor analysis. All statistical analyses were performed using SPSS 27.0 (IBM Corporation, Armonk, New York, United States).

**Table 1:** Characteristics of the lung transplant recipient patients during the COVID-19 pandemic from 1 December 2022 to 28 February 2023.

Covariate	All patients			Group comparison		
	(*N* = 52)	LTx recipients not infected by SARS-CoV-2 (*n* = 9)	LTx recipients with asymptomatic SARS-CoV-2 infection (*n* = 2)	LTx recipients with mild-to-moderate COVID-19 (*n* = 32)	LTx recipients with severe-to-critical COVID-19 (*n* = 9)	*P*-value
Sex, male/female	48/4	7/2	2/0	30/2	9/0	<0.001
Age at COVID-19 diagnosis, years	51.71 ± 11.67	53.44 ± 8.41	41.00 ± 0.00	48.34 ± 10.56	64.33 ± 10.44	0.001
Age at COVID-19 diagnosis ≥65 years	7 (13.5%)	1 (11.1%)	0 (0.0%)	0 (0.0%)	6 (66.7%)	<0.001
Time after transplant, months	33.87 ± 25.9	19.44 ± 14.31	76.00 ± 11.31	36.75 ± 27.25	28.67 ± 21.45	0.027
Transplant procedure						0.918
Bilateral	4 (7.7%)	0 (0.0%)	0 (0.0%)	3 (9.4%)	1 (11.1%)	
Unilateral left lung	21 (40.4%)	3 (14.3%)	1 (50.0%)	14 (43.8%)	3 (14.3%)	
Unilateral right lung	27 (51.9%)	6 (66.7%)	1 (50.0%)	15 (46.9%)	5 (55.6%)	
Underlying disease						0.063
Occupational pneumoconiosis	29 (55.8%)	5 (55.6%)	2 (100.0%)	21 (65.6%)	1 (11.1%)	
Emphysema	12 (23.1%)	2 (22.2%)	0 (0.0%)	7 (21.9%)	3 (33.3%)	
Pulmonary fibrosis/interstitial lung disease	11 (21.2%)	2 (22.2%)	0 (0.0%)	4 (12.5%)	5 (55.6%)	
Comorbidities						
Glomerular filtration rate ≤30 ml/min/1.73 m^2^	8 (15.4%)	1 (11.1%)	0 (0.0%)	4 (12.5%)	3 (33.3%)	0.404
Diabetes	18 (34.6%)	3 (33.3%)	0 (0.0%)	10 (31.3%)	5 (55.6%)	0.396
Pre-existing chronic lung allograft dysfunction	11 (21.2%)	0 (0.0%)	0 (0.0%)	5 (15.6%)	6 (66.7%)	0.002
Hypertension	3 (5.8%)	0 (0.0%)	0 (0.0%)	0 (0.0%)	3 (33.3%)	0.002
Cardiovascular disease	6 (11.5%)	0 (0.0%)	0 (0.0%)	3 (9.4%)	3 (33.3%)	0.123
Immunosuppression						-
Tacrolimus	50 (96.2%)	9 (100.0%)	2 (100.0%)	30 (93.8%)	9 (100.0%)	
Tacrolimus trough concentration before the reopening policy	9.09 ± 2.62	10.58 ± 2.81	7.20 ± 0.57	8.27 ± 2.62	9.48 ± 2.19	0.181
Cyclosporine	2 (3.8%)	0 (0.0%)	0 (0.0%)	2 (6.3%)	0 (0.0%)	
Cyclosporine trough concentration before the reopening policy		—	—	235.40 ± 103.24	—	
Proliferation signal inhibitor	51 (98.1%)	9 (100.0%)	2 (100.0%)	31 (96.9%)	9 (100.0%)	
Prednisone	52 (100.0%)	9 (100.0%)	2 (100.0%)	32 (100.0%)	9 (100.0%)	
Vaccination status						0.011
None	39 (75.9%)	7 (77.8%)	1 (50.0%)	22 (68.8%)	9 (100.0%)	
Partial immunization	10 (19.2%)	1 (11.1%)	0 (0.0%)	9 (28.1%)	0 (0.0%)	
Full immunization	2 (3.8%)	0 (0.0%)	1 (50.0%)	1 (3.1%)	0 (0.0%)	
Booster immunization	1 (1.9%)	1 (11.1%)	0 (0.0%)	0 (0.0%)	0 (0.0%)	
Blood type (%)						0.139
A	16 (30.8%)	2 (22.2%)	0 (0.0%)	12 (37.5%)	2 (22.2%)	
B	19 (36.5%)	3 (33.3%)	0 (0.0%)	13 (40.6%)	3 (33.3%)	
O	14 (26.9%)	4 (44.0%)	2 (100.0%)	6 (18.8%)	2 (22.2%)	
AB	3 (5.8%)	0 (0.0%)	0 (0.0%)	1 (3.1%)	2 (22.2%)	

## 3 Results

### 3.1 Study population and baseline characteristics

A total of 52 LTx recipients were included in this study, and the baseline characteristics of the complete study population stratified by disease severity are presented in Table 1. The recipients comprised 48 men and 4 women, with an average age of 51.71 ± 11.67 years. The mean interval between transplantation and the commencement of this study on 1 December 2022 was 33.87 ± 25.96 months for the overall group, whereas the mean intervals for the community- and hospital-acquired cases were 35.50 ± 26.25 months and 15.60 ± 8.88 months, respectively, showing a significant difference (*P* = 0.001). The underlying diseases among LTx recipients included occupational pneumoconiosis (29/52, 55.77%), idiopathic pulmonary fibrosis (11/52, 21.15%), chronic obstructive pulmonary disease (11/52, 21.15%), and Kartagener syndrome (1/52, 1.92%). For the hospital-acquired cases, five patients were admitted following complications after the lung transplantation: one for airway complications, one for acute rejection, two for pneumonia, and one for heart failure. The predominant immunosuppressive regimen was tacrolimus + mycophenolate mofetil/mycophenolate sodium + prednisone (48/52; 92.31%). Only 3.85% (2/52) of the study population received two doses of COVID-19 vaccine, and both had mild COVID-19. Among the LTx recipients, 9 remained uninfected, while 43 were infected, including 2 asymptomatic cases, 26 mild cases, 6 moderate cases, and 9 severe or critical cases. The prevalent variant during the study period in China was the Omicron variant (8). During this timeframe, from the declaration of reopening to the end of the study, no LTx surgeries were conducted at our center.

Among LTx recipients who remained uninfected by SARS-CoV-2, those with asymptomatic SARS-CoV-2 infection, mild-to-moderate COVID-19, and severe-to-critical COVID-19 exhibited significant differences in sex proportion, age, time after transplantation, pre-existing chronic lung allograft dysfunction, and hypertension based on univariate analysis results (Table 1). Logistic analysis showed that age >65 years at the time of COVID-19 diagnosis and pre-existing chronic lung allograft dysfunction were risk factors for LTx severe-to-critical COVID-19 [odds ratios (OR) = 84.0, 95% confidence interval (CI) 74.474–944.116 and OR = 15.2, 95% CI: 2.860–80.774, respectively].

### 3.2 Clinical manifestations and laboratory findings of LTx recipients at COVID-19 diagnosis

The clinical manifestations and laboratory findings of LTx recipients at the time of COVID-19 diagnosis are detailed in Table 2. Among infected LTx recipients, the predominant clinical symptoms included fever (69.76%, 30/43), cough (72.09%, 31/43), and shortness of breath (55.81%, 24/43). SARS-CoV-2-infected LTx recipients were treated in outpatient or in-hospital settings depending on their need for oxygen, respiratory distress, and pneumonia on imaging, and decisions on disease management were made by their attending physicians. The proportion of inpatients aged >65 years was significantly higher than that of outpatients (*P* = 0.001). Regarding clinical manifestations, the proportion of hospitalized patients with fever was significantly higher than that of outpatients (*P* = 0.008). In addition, the proportion of patients with abnormal chest imaging in hospitalized patients with COVID-19 was significantly higher than that of outpatients (*P* = 0.000).

**Table 2:** Clinical manifestations and laboratory findings of LTx recipients at COVID-19 diagnosis during the COVID-19 pandemic from 1 December 2022 to 28 February 2023.

Covariate	All patients (*n* = 43)	Group comparison		
		Outpatients (*n* = 27)	Hospitalized patients (*n* = 16)	*P*-value
Sex, male/female	41/2	25/2	16/0	0.265
Age >65 years (%)	6 (14.0%)	0 (0.0%)	6 (37.5%)	0.001
Source of infection				
Community-acquired	38 (88.4%)	27 (71.1%)	11 (28.9%)	
Hospital-acquired	5 (11.6%)	0 (0.0%)	5 (100.0%)	
Presenting symptoms (%)				
Fever	30 (69.8%)	15 (50.0%)	15 (50.0%)	0.008
Cough	31 (72.1%)	12 (38.7%)	19 (61.3%)	0.744
Shortness of breath	24 (55.8%)	12 (50.0%)	12 (50.0%)	0.051
Abnormal chest imaging	12 (27.9%)	1 (8.3%)	11 (91.7%)	<0.001
Lowest PaO_2_/FiO_2_		—	197.6 ± 20.4	
Laboratory findings		—		
Lowest leukocyte count ×10^9^/L		—	6.91 ± 0.80	
Lowest absolute lymphocyte count ×10^9^/L		—	0.77 ± 0.12	
Highest LDH (U/L)		—	313.8 ± 24.1	
Highest IL-6 (pg/ml)		—	308.5 ± 236.7	
Highest CRP (mg/L)		—	63.2 ± 10.4	
Highest serum creatinine µmol/L		—	166.6 ± 21.3	
Therapy				
Paxlovid		—	2 (12.5%)	
Azvudine		—	11 (68.8%)	

CRP, C-reactive protein; IL, interleukin; LDH, lactate dehydrogenase.

**Figure 1:**
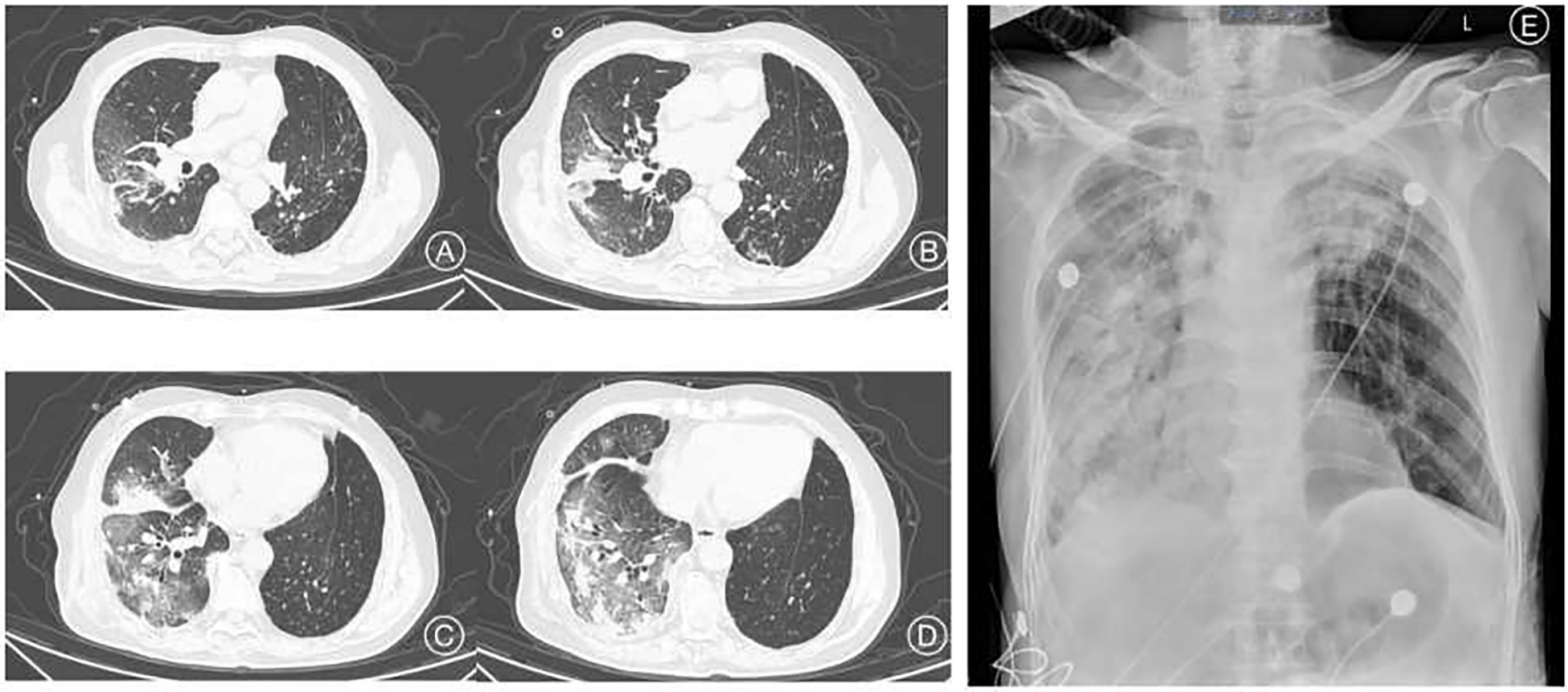
Clinical imaging of a 69-year-old man with pneumoconiosis, diagnosed before LTx and who underwent right single lung transplant surgery one and a half years before the COVID-19 episode. Chest CT images (**A**–**D**) show infiltration mainly involving the right transplanted lung. Chest radiograph image (**E**) 5 days after the CT evaluation. CT, computed tomography.

**Figure 2:**
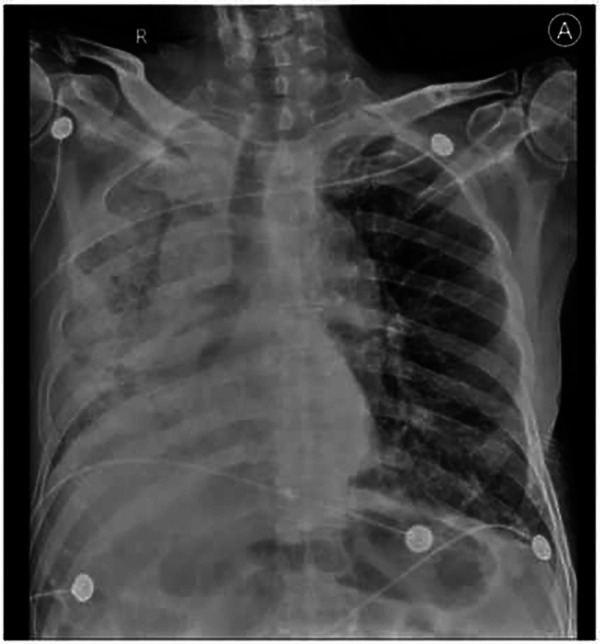
Clinical imaging of a 62-year-old man diagnosed with IPF before LTx and who underwent single-LTx surgery 2 years before COVID-19. Chest radiograph image shows massive consolidation of the allograft (right lung). IPF, idiopathic pulmonary fibrosis.

Among the 43 infected patients, 37.21% (16/43) were hospitalized after assessment. All hospitalized patients underwent chest imaging examinations, and 11 (91.7%) had manifestations of COVID-19 pneumonia. Notably, chest images of unilateral LTx recipients revealed different involvement of the allograft and native lungs. Specifically, 71.43% (10/14) of unilateral LTx recipients predominantly experienced pneumonia affecting the native lung (Figure 1), whereas 14.29% (2/14) had pneumonia primarily affecting the allograft (Figure 2).

### 3.3 Therapeutic management and clinical outcomes of LTx recipients with COVID-19

In total, 43 LTx recipients infected with SARS-CoV-2 were clinically classified as asymptomatic SARS-CoV-2 infection (2/43, 4.65%), mild-to-moderate COVID-19 (32/43, 74.42%), and severe-to-critical COVID-19 (9/43, 20.39%). Individuals with asymptomatic SARS-CoV-2 infection and mild COVID-19 did not receive small-molecule antiviral drugs. Four of the patients with moderate COVID-19 received azvudine treatment; two and seven patients with severe-to-critical COVID-19 received nirmatrelvir/ritonavir treatment (2/9, 22.22%) and azvudine treatment (7/9, 77.78%), respectively. Among LTx recipients with severe-to-critical COVID-19, 22.22% (2/9) received conventional oxygen therapy, 22.22% (2/9) received non-invasive positive pressure ventilation or high-flow nasal oxygen therapy, and 55.56% (5/9) received invasive ventilation.

All of the 34 LTx recipients with asymptomatic SARS-CoV-2 infection and mild-to-moderate COVID-19 fully recovered, while 6 LTx recipients with severe-to-critical COVID-19 died, accounting for 66.67% (6/9) of this severe and critical group, 13.95% (6/43) of the SARS-CoV-2 infection group, and 11.54% (6/52) of all LTx recipients in this study.

Among the 16 hospitalized LTx recipients with COVID-19, 10 survived on the 28th day and 6 died. Upon comparing the age, clinical types, community-acquired or hospital-acquired infections, minimum oxygenation index during hospitalization, minimum total white blood cell count, and a few other variables between the surviving and deceased groups, only clinical types and minimum PO_2_/FiO_2_ during hospitalization significantly differ between these two groups (Table 3).

**Table 3:** Clinical manifestations and laboratory findings of hospitalized LTx recipients during the COVID-19 pandemic from 1 December 2022 to 28 February 2023.

Covariate	All hospitalized patients	Group comparison		
	(*n* = 16)	Patients alive at 28 days (*n* = 10)	Patients deceased by 28 days (*n* = 6)	*P*-value
Sex (male)	16	10	6	
Age >65 years (%)	6 (37.5%)	3 (30.0%)	3 (50.0%)	0.424
Source of infection				0.790
Community-acquired	10 (62.5%)	6 (60.0%)	4 (40.0%)	
Hospital-acquired	6 (37.5%)	4 (66.7%)	2 (33.3%)	
LTx recipients with severe-to-critical COVID-19	95 (6.3%)	3 (30.0%)	6 (60.0%)	0.006
Lowest PaO_2_/FiO_2_		232.7 ± 18.0	139.2 ± 36.0	0.021
Laboratory findings				
Lowest leukocyte count ×10^9^/L		6.3 ± 0.7	7.9 ± 1.8	0.372
Lowest absolute lymphocyte count ×10^9^/L		0.9 ± 0.2	0.6 ± 0.2	0.203
Highest LDH		286.5 ± 25.9	359.3 ± 44.2	0.149
Highest IL-6		83.1 ± 70.9	684.2 ± 622.9	0.381
Highest CRP		59.1 ± 15.1	70.1 ± 12.5	0.622
Highest serum creatinine µmol/L		143.2 ± 18.4	205.7 ± 46.1	0.162
Antivirus medication				0.117
Nirmatrelvir/ritonavir	2 (12.5%)	2 (100.0%)	0 (0.0%)	
Azvudine	11 (68.8%)	5 (45.5%)	6 (54.5%)	
None	3 (18.8%)	3 (100.0%)	0 (0.0%)	

CRP, C-reactive protein; IL, interleukin; LDH, Lactate dehydrogenase.

## 4 Discussion

This study aimed to investigate the clinical manifestations and prognosis of LTx recipients infected with SARS-CoV-2 during the COVID-19 pandemic. SARS-CoV-2 can infect any person; however, severe and unfavorable outcomes have been most notable among vulnerable populations. Since the beginning of the COVID-19 pandemic, vulnerable populations have always been a priority in epidemic prevention, control, and treatment. Transplant recipients may be at a higher risk of infection by SARS-CoV-2 because of the baseline use of immunosuppressants, underlying comorbidities, and frequent contact with healthcare workers. At the beginning of the COVID-19 pandemic, the clinical aspects and impact of COVID-19 on SOT recipients remained unclear. Early studies reported mortality rates ranging from 7.1% to 53% (10, 11). In Spain, a nationwide study reported that the incidence of COVID-19 in SOT recipients was twofold higher than that in the general population. Mortality differed significantly across the transplant types, with the probability of death being significantly higher in LTx recipients than in other transplant recipients. Lung transplantation is a risk factor for mortality (12). The results of a large, prospective, multicenter study of SOT recipients with COVID-19 revealed that mortality among hospitalized SOT recipients was around 20%, which was generally higher than that in the general population. When the thoracic organs were restricted to lung transplantation only, the results of the analysis between the thoracic and non-thoracic organs and mortality were similar (13).

Between 26 September 2022 and 2 March 2023, the Chinese Center for Disease Control and Prevention received 29,248 valid genome sequences of COVID-19, all identified as Omicron variants. The predominant epidemic strains were BA.5.2.48 (54.0%), BF.7.14 (25.6%), and BA.5.2.49 (12.8%) (14). During the Omicron era, our study showed that 82.69% of LTx recipients in this study were infected with SARS-CoV-2, while 17.31% had severe and critical COVID-19 during the study period. Furthermore, some LTx recipients continued to undergo strict self-isolation, leading to nine individuals remaining uninfected. Based on clinical relevance and in accordance with previous studies, we assessed the risk factors for severe and critical COVID in LTx recipients. The average age of LTx with severe and critical COVID-19 was significantly higher than that of other clinical types of SARS-CoV-2 infections, including asymptomatic SARS-CoV-2 infection and mild-to-moderate COVID-19, as defined by the Diagnosis and Treatment Plan for COVID-19 (Tenth Edition) issued by the National Health Commission of the People's Republic of China. In addition, the proportion of severe and critical COVID-19 patients over 65 years of age was significantly higher than that of other clinical types of SARS-CoV-2 infections. Statistical analysis also showed that LTx recipients with chronic transplantation lung dysfunction had a significantly increased risk of developing severe and critical diseases after infection. However, the data did not suggest that blood type, single- or double-lung transplantation procedure, underlying lung diseases before transplantation, time after lung transplantation, tacrolimus trough concentration before the reopening policy, glomeruar filtration rate (GFR), or diabetes increased the risk of severe and critical COVID (15). We observed five hospital-acquired COVID-19 cases, and their mean interval from transplantation to the commencement of this study significantly differed from that of community-acquired cases.

Regarding clinical manifestations of infected LTx recipients, the most interesting finding was the difference between the imaging manifestations of the transplanted and autologous lungs in single-LTx recipients with COVID-19 pneumonia. COVID-19 pneumonia affecting only the native lung after one-lung transplantation has been reported (16); however, the reason for this has not been elucidated. Histopathological examination of a patient who died of COVID-19 revealed interstitial and perivascular, predominantly lymphocytic, pneumonia with multifocal endothelialitis. When SARS-CoV-2 invades the human respiratory tract, the receptor-binding domain (RBD) of the S protein on the viral surface recognizes the host cell receptor angiotensin-converting enzyme 2 (ACE2) and binds to it to enter target cells (17, 18). Published data reveal significantly greater numbers of ACE2-positive cells in the lungs of patients with COVID-19 and influenza than in uninfected controls (19). Studies have also shown that compared with controls, the lung tissue of patients who died of COVID-19 had upregulated expression of proteins such as NPC1/2, CEACAM1, CTSL, EIF4E, and PABPN1, which involve virus-binding receptors, proteases, host mRNA degradation, and translation cessation. However, there were no statistically significant differences in the expression of ACE2, CD209, or CLEC4M proteins (20).

In general, the treatment for COVID-19 in SOT recipients is similar to that in the general population and is based on antiviral and anti-inflammatory medications (21). Notably, there are no randomized clinical trials (RCT) of COVID-19 treatments that focus on the SOT population. Therefore, the data supporting their use in this specific high-risk population are extrapolated from clinical trials in general non-transplant patients and from real-world experiences described in a multitude of retrospective studies in SOT recipients that have been published to date (22). Aggressive treatment of patients with SOT is highly recommended because immunosuppressed patients have a well-described blunted antibody response to infection and remain at a high risk of severe outcomes. The China National Health Commission recommends nirmatrelvir/ritonavir and molnupiravir to treat adult patients with mild-to-moderate symptoms and high-risk factors for progression to severe illness within 5 days of onset, azvudine tablets for adult patients with moderate novel coronavirus infection, and molnupiravir capsules to be used within 5 days of onset in adult patients with mild-to-moderate symptoms or in patients with high-risk factors for progression to severe COVID-19. For critically ill patients, even if the onset of the disease exceeds 5 days, because of the high nucleic acid load (cycle threshold value < 30), the recommended drugs mentioned above can still be prudently considered for treatment (23). From 1 December 2022 to 28 February 2023, nirmatrelvir/ritonavir and azvudine were clinically accessible but not sufficient for the vigorously increasing cases in this study; thus, none of the outpatient COVID-19 LTx recipients received antiviral drugs, and extremely limited number of the hospitalized COVID-19 LTx recipients received nirmatrelvir/ritonavir. The lack of treatment drugs to prevent COVID-19 diagnosed population with a high-risk factor to prevent COVID-19 progressing may be related to the relatively high proportion of severely critical COVID-19 cases in this group of LTx recipients, which was 17.31% (9/52). There are limited data on antiviral therapy for severe COVID-19 and even fewer data on SOT recipients with severe COVID-19. The efficacy and safety of Paxlovid (nirmatrelvir/ritonavir) in hospitalized adult patients with SARS-CoV-2 (Omicron BA.2.2 variant) infection and severe comorbidities were studied by Liu et al., who found that Paxlovid showed no significant reduction in the risk of all-cause mortality on day 28 and the duration of SARS-CoV-2 RNA clearance in hospitalized adult COVID-19 patients with severe comorbidities (24). Concerning the clinical benefits of the anti-inflammatory therapy, based on the clinical trial RECOVERY and REMAP Trial, dexamethasone was endorsed for use in hospitalized patients with COVID-19 who required oxygen therapy, including oxygen supplementation, high-flow nasal cannula to mechanical ventilation or extracorporeal membrane oxygenation; however, there were no adequate clinical trials available to evaluate the efficacy of dexamethasone for severe COVID-19 in the SOT population (25, 26). There are concerns about the safety of dexamethasone use among LTx recipients, and in this study, the baseline dose of prednisone was maintained during treatment. Although the survival benefit of baricitinib and tocilizumab has been reported in the general population (27, 28), data on their use in transplant recipients are limited. A matched cohort study was conducted to evaluate the safety and efficacy of tocilizumab for treating severe respiratory symptoms due to COVID-19, particularly among SOT recipients. Tocilizumab appeared safe but was not associated with decreased 90-day mortality (29). In this study, neither baricitinib nor tocilizumab was administered to LTx recipients owing to no definite recommendation on this subject or concerns about secondary infections (30, 31).

Our study had the following limitations. First, although this study reported the detailed characteristics and clinical outcomes of SARS-CoV-2 infection in all surviving LTx recipients followed up at our center during the study period, the limited number of cases was an intrinsic limitation of this single-center report. Finally, the accessibility of clinical medication was influenced by the complexity of the situation, such as an insufficient supply chain of medicine during the pandemic, which potentially affected the clinical outcomes of COVID-19 LTx recipients in this study.

In conclusion, LTx recipients in this cohort exhibited a notable susceptibility to SARS-CoV-2, with 82.69% of individuals diagnosed with COVID-19. Data from this study revealed that the mortality rate among the severe and critical patients was as high as 66.67% (6/9). From the sequential follow-up of these patients, it was found that SARS-CoV-2 infection not only affected the short-term prognosis of LTx recipients after infection but also had potential impacts on secondary infection after the acute course of COVID-19, lung function, and quality of life in the long-term. Further research describing the long-term effects on LTx recipients is necessary to treat and manage these LTx recipients to avoid loss of graft function and improve their survival and quality of life.

## Data Availability

The raw data supporting the conclusions of this article will be made available by the authors without undue reservation.
